# Gastrointestinal Nematode Control in Lithuanian Sheep Farms: Insights from a Questionnaire Survey

**DOI:** 10.3390/ani15111542

**Published:** 2025-05-24

**Authors:** Agnė Beleckė, Tomas Kupčinskas, Inga Stadalienė, Rasa Adomkienė, Saulius Petkevičius

**Affiliations:** Department of Veterinary Pathobiology, Laboratory of Parasitology, Faculty of Veterinary Medicine, Lithuanian University of Health Sciences, Tilžės 18, LT-47181 Kaunas, Lithuania; tomas.kupcinskas@lsmu.lt (T.K.); inga.stadaliene@lsmu.lt (I.S.); rasa.adomkiene@lsmu.lt (R.A.); saulius.petkevicius@lsmu.lt (S.P.)

**Keywords:** anthelmintic resistance, gastrointestinal nematodes, sheep farming, survey, worm control

## Abstract

Anthelmintic resistance (AR) in gastrointestinal nematodes is a growing global concern that threatens effective parasite control in livestock farming. This study aimed to assess gastrointestinal nematode (GIN) control practices and identify risk factors associated with AR development on Lithuanian sheep farms. A cross-sectional survey was conducted between 2022 and 2023 among 71 members of the Lithuanian Sheep Breeders Association, covering farm management, grazing, and deworming practices. Anthelmintics were used by 90.2% of farmers, with most treating their flocks twice a year. Only 18.3% of farmers performed parasitological testing to guide treatments, with significantly more organic farms applying this approach than traditional farms. Treatment frequencies were significantly higher in traditional farms. Most farmers (45.1%) relied on the visual appraisal of sheep weight for dosing, while 35.2% dosed according to the weight of a medium-sized animal. Macrocyclic lactones were the most commonly used anthelmintics (50.7%). Quarantine procedures for new animals were implemented by less than half of the farmers (44.4%), while others treated without isolation (56.6%). These findings indicate reliance on non-strategic parasite control methods and limited use of diagnostics, highlighting the need for improved farmer education to promote sustainable parasite control and reduce AR risk in Lithuanian sheep farms.

## Simple Summary

Gastrointestinal parasite infections are a major challenge in sheep farming, affecting animal health, welfare, and farm productivity. Anthelmintic drugs, commonly used to control these parasites, can become less effective when used too frequently or without proper diagnostic guidance, leading to the development of resistance. This study assessed parasite control practices on Lithuanian sheep farms and explored factors that may promote resistance. A survey of 71 farmers showed that most used anthelmintics routinely, mainly twice per year, but only a small proportion based treatment decisions on diagnostic testing. Organic farms were more likely to use diagnostic methods than traditional farms. Many farmers relied on the visual estimation of sheep weight rather than accurate weighing when determining dosages, increasing the risk of underdosing. Macrocyclic lactones were the most commonly used type of anthelmintic. Quarantine measures for newly introduced animals were not consistently applied. These findings highlight a heavy reliance on routine, non-strategic treatments, and limited use of evidence-based approaches. Strengthening farmer education on parasite management, promoting regular diagnostic testing, accurate dosing, and effective quarantine procedures are essential steps to slow the development of anthelmintic resistance and to support sustainable sheep farming practices.

## 1. Introduction

Gastrointestinal nematode (GIN) infections are a major issue affecting the health and productivity of small ruminants globally. These parasites cause significant economic losses due to reduced growth rates, decreased wool production, and impaired reproductive efficiency [1,2,3]. In response, the widespread use of anthelmintics has been the primary strategy for nematode control. However, over-reliance on these drugs has led to the emergence of anthelmintic resistance (AR), which poses a growing threat to sheep farming sustainability [1,4]. Nematode resistance to benzimidazoles (BZ), levamisole (LEV), ivermectin (IVM), and other macrocyclic lactones (ML) is becoming increasingly widespread globally, with resistance expanding in many parts of Europe. However, knowledge gaps remain in certain regions, particularly in Eastern Europe [3,5]. This region has a limited number of registered anthelmintics [3]. The average prevalence of resistance at the farm level was found to be 86% for BZ, 52% for ML excluding moxidectin (MOX), 48% for LEV, and 21% for MOX [5]. Multiple cases of AR in GIN of sheep have been identified in France, Greece, and Italy [6]. Additionally, multidrug resistance (MDR) has been documented in the UK and Ireland, and it is becoming an increasing Scotland [7,8,9] and France [10].

Questionnaire-based surveys on worm control practices in small ruminants have previously been conducted in Denmark [11,12,13], the United Kingdom [14,15,16], France [17], Slovakia [18], Norway [19,20], Sweden [21,22], and Lithuania [23]. Helminth control strategies must now include management practices that focus on extending the effectiveness of anthelmintics. This is especially crucial given the low likelihood of new active compounds being introduced in the near future, and the fact that commercially available subunit antihelminth vaccines are still a long way off [24]. It is now evident that in order to ensure long-term profitability in livestock production, sustainable parasite control programs must incorporate integrated novel non-chemical methods, enhanced pasture management and husbandry practices, along with strategies that manage refugia [1]. For the best practices to be effectively implemented, it is essential that farmers receive proper guidance on parasite management strategies, with a focus on the strategic use of anthelmintics. Implementing integrated parasite control strategies that rely less on chemical anthelmintics is complex due to various factors, including differences in local parasite species, host breeds, climate, management practices, and traditions. Additionally, regional and farm-specific drug resistance further complicates the situation. The lack of data on the best use of anthelmintics to balance animal health, productivity, and resistance prevention adds to these challenges [25].

There is currently a lack of comprehensive data on worm control practices and livestock management on sheep farms in Lithuania. Gaining knowledge in this area is essential for developing effective guidelines to prevent anthelmintic resistance. Therefore, the objective of this study was to expand our knowledge of worm control practices on sheep farms in Lithuania.

## 2. Materials and Methods

This study utilized a cross-sectional questionnaire survey to collect data on the farming practices of members of the Lithuanian Sheep Breeders Association. The survey was conducted over a two-year period from 2022 to 2023. The participants in this study were all members (193) of the Lithuanian Sheep Breeders Association, which encompasses the entire territory of Lithuania. Data collection was performed via telephone interviews with each member. The interviews were conducted by the authors themselves. Of the 193 members contacted, 71 participants completed the questionnaire (response rate: 36.8%). Among the remaining individuals, 73 did not answer the phone despite repeated call attempts, and 49 explicitly refused to participate in the survey. Verbal informed consent was obtained prior to each interview, followed by the mailing of a written consent form to all participants for documentation purposes.

The questionnaire consisted of 16 questions (Supplementary Materials) and was developed specifically for this study. It included 9 multiple-choice and 7 open-ended items, designed to collect detailed information on key aspects related to parasite control in sheep farms. The questionnaire covered the following thematic areas: (1) Farm characteristics (4 questions); (2) Grazing practices (3 questions), focusing on the duration and system of grazing; and (3) Worm-control practices (9 questions), addressing the timing and frequency of treatments, types and dosages of anthelmintic products used, and quarantine measures applied for newly introduced animals.

Data were analyzed using “SPSS for Windows version 20”. Descriptive statistics were employed to summarize the data. To provide a measure of precision around proportions, 95% confidence intervals (CI) were calculated for categorical variables. To assess statistical significance, Chi-square tests were used to compare proportions between categorical variables. When expected cell frequencies were less than five, Fisher’s Exact Test was applied to ensure statistical validity. Comparisons of continuous variables, including average treatment frequency, between traditional and organic farms were performed using independent samples *t*-tests. A *p*-value of less than 0.05 was considered statistically significant.

## 3. Results

### 3.1. General Information

**Figure 1:**
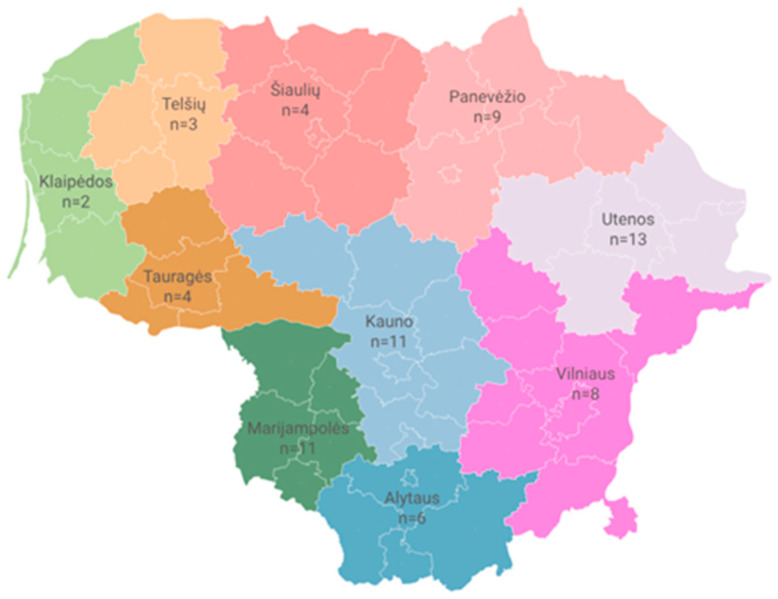
Regional distribution of surveyed sheep farms across Lithuania’s 10 counties.

During 2022–2023, all 193 members of the Lithuanian Sheep Breeders Association, geographically distributed across all 10 counties of Lithuania, were surveyed, with the highest proportion of respondents from Utena County (13/71, 18.31%), followed Kaunas and Marijampolė Couties (11/71, 15.49% each) (Figure 1). The questionnaire was completed by 71 members, resulting in a response rate of 36.8% (95% CI: 29.9–44.1%).

Among the respondents, 60.6% of farms were organic and 39.4% were traditional (95% CI: 48.8–71.5% and 28.5–51.2%, respectively). The average flock size across these farms was approximately 184.5 sheep (range: 10–1600). Rotational grazing was practiced on 36.6% (*n* = 26, 95% CI: 25.6–48.7%) of the farms surveyed, while 63.4% (*n* = 45, 95% CI: 51.3–74.4%) kept sheep on a single pasture throughout the grazing period. The predominant sheep breed was Lithuanian Black Head, present in 46.5% (*n* = 33, 95% CI: 34.9–58.3%) of farms, followed by mixed breeds (18.3%), Merinolandschaf (5.6%) and Suffolk (5.6%). Grazing periods began primarily in May (*n* = 34), followed by April (*n* = 30) and March (*n* = 7), and ended mostly in November (*n* = 54), with fewer in December (*n* = 16) and October (*n* = 1). Most farms (87.3%, 95% CI: 77.9–93.9%) did not keep other animals. Among those that did, 8.5% kept goats, 2.8% cattle, and 1.4% horses. The average grazing area was 34.51 hectares, ranging from 1 to 200 hectares. Water supply methods varied: 67.6% (*n* = 48, 95% CI: 55.3–78.2%) of farms used well water, while the rest relied on access to natural moist areas in pastures combined with other on-farm water sources.

### 3.2. GIN Treatment Strategies

Only 18.3% (*n* = 13, 95% CI: 10.1–29.1%) of farmers used parasitological analysis of fecal samples as an indicator for treatment effectiveness. Organic farms were more likely to utilize this method, with 23.3% (*n* = 10, 95% CI: 12.4–37.2%) conducting parasitological tests, compared to only 10.7% (*n* = 3, 95% CI: 2.3–28.2%) of traditional farms (*p* < 0.05). The main factors influencing decisions on when to treat against helminths were previous experience (38.0%) and the occurrence of diarrhea (22.5%). The spring before turn-out and autumn before housing were the most common times for sheep treatment. It was estimated that 90.2% of sheep farmers (*n* = 64, 95% CI: 81.4–95.7%) used anthelmintics to control gastrointestinal nematodes. Based on the questionnaire results, lambs were drenched on average 1.54 times per year, and yearlings/ewes 1.49 times per year. Annual treatment frequencies showed that most farms (49.3%) drenched their sheep twice per year, 38.0% treated once per year, and 4.2% treated three times per year. Additionally, 8.5% of farmers reported that they do not treat their sheep at all. A few farmers treated lambs (8.5%) and yearlings/ewes (4.2%) more than twice a year (Table 1). No statistically significant differences were observed between farm types in annual treatment frequency (*p* > 0.05).

In comparing treatment frequencies between traditional and organic farms, notable differences were observed. The average drenching rate for yearlings/ewes in traditional farms was 1.71 treatments per year, which is approximately 1.27 times higher than the 1.35 treatments per year observed in organic farms. This difference was statistically significant (*p* < 0.05). Similarly, the treatment rate for lambs in traditional farms was 1.71 treatments per year, approximately 1.19 times higher than the 1.44 treatments per year in organic farms, which was also statistically significant (*p* < 0.05). For anthelmintic dose estimation, most farmers (45.1%) relied solely on the visual appraisal of sheep weight. Meanwhile, 35.2% weighed a medium-sized animal and dosed the whole flock based on that weight (Table 2).

ML were the most frequently used anthelmintic class, reported by 50.7% of farms. BZ were used by 11.3% of farms (Table 3). Rotation between BZ and ML was practiced in 16.9% of farms. Furthermore, 12.3% of farmers reported purchasing new sheep from abroad. However, less than half (44.4%) implemented quarantine measures upon arrival, while 56.6% opted only to drench newly purchased sheep without quarantine. The difference between implementing quarantine and drenching without quarantine was statistically significant (*p* < 0.05).

## 4. Discussion

GIN infections present a significant challenge to sheep farming globally, and Lithuania is no exception. The results of this study provide important insights into the worm control practices on Lithuanian sheep farms. The response rate of 36.8% from the Lithuanian Sheep Breeders Association members provides a substantial dataset, allowing for the characterization of current practices and the identification of potential areas for improvement. However, the relatively low response rate may be attributed to several factors, such as limited farmer engagement in scientific surveys, lack of time or interest, and possible concerns regarding the confidentiality of shared information. Additionally, some farmers may not perceive gastrointestinal nematode infections as a pressing issue, which could further reduce their motivation to participate.

The study found that 36.6% of the farms practiced rotational grazing, while a majority, 63.4% kept their sheep on a single pasture with a shelter throughout the grazing period. The minority of respondents (12.7%) practiced mixed or alternate grazing with cattle or horses, a well-known strategy for reducing parasite pressure and minimizing the reliance on anthelmintics for parasite management [26].

The findings indicate the limited use of parasitological diagnostics among sheep farmers, particularly in traditional farms, where treatment decisions were less evidence-based. Higher drenching frequencies observed in traditional farms suggest a more intensive, possibly prophylactic approach, which may increase the risk of developing anthelmintic resistance. Only 18.3% of farmers utilized parasitological analysis of fecal samples to guide treatment, while the majority relied on past experiences (38.0%) or visible symptoms like diarrhea (22.5%), similar to reports from several European countries [15,19,27]. In Belgium, only few sheep farmers (9%) regularly used fecal egg counts to monitor worm infections [28]. This dependence on subjective indicators rather than objective diagnostic methods highlights a critical area for improvement in worm control practices in Lithuania. Greater access to and use of fecal egg count monitoring could enable more targeted treatments, potentially reducing anthelmintic use and slowing the development of AR. According to a study in Norway [19], 90% of farmers did not suspect treatment inefficacy, so changing established practices might not be seen as necessary. Farmers may undervalue diagnostic testing if their treatment routines yield acceptable results, viewing it as an unnecessary expense. With multiple farming challenges like time management, livestock welfare, and regulatory compliance, parasitological testing may not be a top priority, especially if anthelmintic treatment seems effective. Additionally, a lack of encouragement from veterinarians could contribute to low testing rates.

A prior study by Kupčinskas [23] found that the majority of sheep farmers in Lithuania (92.2%) determined the correct anthelmintic dosage by visually estimating the weight of their sheep. It appears that the situation has improved in recent years, as visual appraisal is now used less frequently (45.1%). Similar results were found in Norway, Sweden, Slovakia, and France, where the visual appraisal of weight was the dominant choice of dose [17,18,20,21]. Incorrect live-weight estimation and improper calibration of drench guns can lead to under-dosing. To ensure proper dosing, it is important to estimate animal weight as accurately as possible, ideally by individually weighing each animal. Alternatively, weighing the heaviest animal and slightly overestimating the dose for all animals can also help ensure correct anthelmintic treatment [20]. In our survey, if weighing each animal or only the heaviest was considered the only acceptable method for dose calculation, only 8.5% of sheep flocks had accurate dose calculations. Burgess et al. [29] suggested that underdosing with substances from the same class might be more critical than drenching frequency, which was found to be low in our survey.

Compared to a previous study [23], the use of anthelmintics in Lithuania has significantly increased from 71.8% to 90.2%, although the drenching rates for lambs and ewes have been increased slightly, from 1.24 to 1.54 and from 1.29 to 1.49 times per year. This drenching rate is slightly lower compared to other European countries. Domke et al. reported mean drenching rates of 2.5 for lambs and 1.9 for ewes in Norway [20], while in Denmark, the rates were 1.9 for lambs and 2.3 for ewes [12]. In the Netherlands, ewes were treated on average 1.53 times per year, while lambs received 2.05 treatments annually [27]. In Belgium, both ewes and lambs were treated more frequently, with mean annual drenching rates of 2.6 and 3.2, respectively [28]. In Scotland and England, the mean drenching rates are higher, ranging from 2.2 to 4.4 treatments per lamb annually [15,30]. The average drenching frequency in France was 5.2 times per year [31], compared to 1.70–1.76 times per year in the Slovak Republic [18]. In Sweden, the use of anthelmintics was relatively low, at 45%, with the majority (76%) of those treatments involving ewes being drenched on a single occasion [21]. Frequent treatments are regarded as a significant risk factor for the development of anthelmintic resistance [32]. Moreover, farmers who treated their ewes more frequently also tended to treat their lambs more frequently [27].

A significant finding from this study is the high prevalence of organic farming (60.6%) compared to traditional systems (39.4%), highlighting the prominence of sustainable practices in Lithuania. Organic farms were more inclined to use parasitological analysis, with 23.3% conducting tests, in contrast to just 10.7% of traditional farms. Variations between traditional and organic farms suggest differing approaches to disease management. The observation that traditional farms drench more frequently than organic farms reflects inherent differences in disease management strategies. Organic farming often emphasizes natural resistance and lower intervention, which is reflected in the lower treatment rates. In contrast, traditional farms, perhaps due to more intensive farming practices, seem to rely more heavily on chemical interventions to manage parasite infection.

ML were the most commonly used anthelmintic class on Lithuanian sheep farms, with 50.7% of farmers reporting their use. This represents a slight decline from the previous study, where ML usage was 56.9% [23]. Similarly, the use of (BZ) also saw a decline, dropping from 15.7% [23] to 11.3%. In contrast, the use of (LEV) showed a modest increase, rising from 3.9% [23] to 5.6%, though it is still used sporadically. This may indicate a potential over-reliance on specific anthelmintic classes, which could accelerate the development of resistance. The choice of anthelmintic drugs in Lithuania appears to align with Northern European countries but differs from other parts of Europe, where there is growing use of ivermectin, along with increased reliance on closantel, doramectin, moxidectin, and combination treatments [3,16,21,23]. Anthelmintic rotation was used only in 23.9% of farms and IVM, BZ, and LEV are the only single substances registered against GIN of sheep in Lithuania. The Nordic–Baltic region has a limited number of registered anthelmintics [3]. However, animal welfare requirements are stringent, and anthelmintics can only be sold with a prescription.

Quarantine routines have been emphasized as a crucial factor in supporting sustainable parasite control and preventing the introduction of AR [19,33]. Farmers in Lithuania prefer to drench new sheep, similar to Norway [19], Sweden [21], and the Netherlands [34]. The trade of livestock both within and between countries appears to play a role in the spread of resistant parasites in Lithuania.

## 5. Conclusions

This study provides valuable insights into worm control practices on Lithuanian sheep farms, identifying key areas for improvement in combating gastrointestinal nematode infections. For instance, most respondents treated their sheep with anthelmintics routinely, without performing a prior diagnosis. Additionally, dose calculations were frequently based on visual estimates, which increases the risk of underdosing. The findings highlight the need for increased education on parasite management, especially for traditional farms. Emphasizing the use of diagnostic tools and the implementation of effective quarantine treatment protocols can help guide better treatment decisions and improve overall parasite control strategies. These measures will be essential in managing anthelmintic resistance and ensuring the long-term sustainability of sheep farming in Lithuania.

## Figures and Tables

**Table 1:** Frequency and distribution of anthelmintic treatments in sheep flocks by age group (*n* = 71).

	Number of Treatments	%	95% CI
Yearlings/ewes	1	38.0%	27.3–49.4%
	2	49.3%	37.9–60.7%
	3	4.2%	0.9–11.7%
	Never	8.5%	3.2–17.5%
Lambs	1	3.2–17.5%	22.9–44.3%
	2	37.8%	27.2–49.4%
	3	8.5%	3.5–16.7%
	Never	7.3%	2.8–15.1%

**Table 2:** Anthelmintic dose estimation methods in the sheep flocks (*n* = 71).

Calculation or Estimation of Weight	%	95% CI
Visual appraisal of weight	45.1%	33.6–56.9%
Weighing a medium sized sheep	35.2%	24.6–47.2%
Weighing the largest sheep	5.6%	1.5–13.6%
Weigh each animal	2.8%	0.3–9.7%
No treatment	11.3%	5.0–21.0%

**Table 3:** Use of anthelmintics against GINs over a three-year period based on farmer responses (*n* = 71), including ML, LEV, BZ, and rotation of anthelmintic classes.

Anthelmintic	%	95% CI
ML	50.7%	39.4–62.0
LEV	5.6%	1.4–11.3
BZ	11.3%	4.2–19.7
BZ + ML	16.9%	8.5–25.4
BZ + ML + LEV	7.0%	1.4–14.1
No treatment	8.5%	2.8–15.5

## Data Availability

The data presented in this study are available on request from the corresponding author.

## Supplementary Materials

The following supporting information can be downloaded at: https://www.mdpi.com/article/10.3390/ani15111542/s1, Questionnaire regarding gastrointestinal parasites in sheep farms in Lithuania.

## Abbreviations

The following abbreviations are used in this manuscript:

AR	Anthelmintic resistance
BZ	Benzimidazoles
CI	Confidence interval
GIN	Gastrointestinal nematodes
IVM	Ivermectin
LEV	Levamisole
ML	Macrocyclic lactones
MOX	Moxidectin
MDR	Multidrug resistance
