# Supplementary material for: Gastrointestinal Nematode Control in Lithuanian Sheep Farms: Insights from a Questionnaire Survey
Source: Animals (Basel). 2025 May 24;15(11):1542. doi: 10.3390/ani15111542 (PMC12153651; doi:10.3390/ani15111542)
Supplement: Supplementary file 1 [file animals-15-01542-s001.zip › animals-3647400-supplementary.pdf]

Survey of Sheep Farmers

Farm ID:
Region:

1 What type of farm do you have?

a Traditional
b Organic

2 Please indicate the approximate number of animals

Sheep	Number
Yearlings/ewes	
Lambs	

3 What breed(s) of sheep do you raise?

4 Other animals kept

	Number		Number		Number
Goats		Cattle		Ohers	

5 Please indicate the approximate area of meadows and sown pastures used for sheep grazing:

	Area (ha)
**a** Permanent pastures (not reseeded for more than 5 years)	
**b** Rotational pastures (reseeded more often than every 5 years)	
**d** Other (e.g., shrubs, forest edges, woodland)	
Toatl area	

6 Please indicate the type of water supply available in the pasture:

a Water troughs (water from a well or pipeline)
b Water troughs (water from a pond, lake, etc.)
c Open water bodies accessible to animals (pond, stream, lake)
d Other (please specify)

7 Please indicate the method of sheep grazing:

a Indoors (barn-based)
b Rotational grazing
c Grazing in the same pasture

Approximate grazing start date:
Approximate grazing end date:

8 Please indicate the main criteria you use to assess gastrointestinal worm infection:

a Fecal sample testing
b Data provided by slaughterhouses
c Diarrhea

d Poor quality/thin coat/wool
e Anemia
f Weight loss/low weight gain/milk yield
g Scientific literature and expert recommendations
h Veterinarian's recommendation
i Advice from another farmer or consultant
j Based on past experience

9 Do you periodically use anthelmintic drugs against parasites?

		Gastrointestinal nematodes	
		Yearlings/ewes	Lambs
Anthelmintic use	Yes (how many times per year)		
	No		

10 Which products do you use to treat gastrointestinal parasites?

	Active ingredient	Product names	
	Benzimidazole	(Panacur, Curaflukes)	
	Levamisole	(Levamisol, Dehelman)	
	Macrocyclic lactones	(Ivermektinas-Bimectin,	
	Kita (nurodyti)		

11 Do you rotate anthelmintic products, and if so, which ones?

Yes	
No	

12 If you use anthelmintics for treatment, please indicate when this is done:

a Before mating/lambing/during dry period
b Immediately after lambing
c In srping, before turn out
d In spring, after turnout
e Before housing
f Before moving to a new pasture
g When clinical signs appear

13 How do you calculate the anthelmintic dose?
a Visual appraisal of weight
b Weighing a medium sized sheep
c Weighing the largest sheep
d Weigh each animal

e No treatment

14 When did you acquire the sheep (excluding local Lithuanian breeds)?

15 Where were the sheep acquired from?

16 Do you quarantine newly acquired animals?

a Yes

b No
